# Synthesis and Characterization of Nanoporous Carbon Carriers for Losartan Potassium Delivery

**DOI:** 10.3390/ma14237345

**Published:** 2021-11-30

**Authors:** Aleksander Ejsmont, Anna Stasiłowicz-Krzemień, Dominika Ludowicz, Judyta Cielecka-Piontek, Joanna Goscianska

**Affiliations:** 1 Faculty of Chemistry, Department of Chemical Technology, Adam Mickiewicz University in Poznań, Uniwersytetu Poznańskiego 8, 61-614 Poznań, Poland; aleksander.ejsmont@amu.edu.pl; 2 Faculty of Pharmacy, Department of Pharmacognosy, Poznań University of Medical Sciences, Święcickiego 4, 61-781 Poznań, Poland; astasilowicz@ump.edu.pl (A.S.-K.); dominika.siakowska@interia.eu (D.L.)

**Keywords:** mesoporous carbon vehicles, chitosan, surface modification, nitrogen doping, adsorption of losartan potassium, drug liberation

## Abstract

Losartan potassium is most commonly used for the treatment of hypertension. In recent years, new applications of this drug have emerged, encouraging the design of novel nanoporous carriers for its adsorption and release. The purpose of this study was to synthesize ordered mesoporous carbon vehicles via a soft-templating method altered with the use of nitrogen precursors and via a hard-templating method followed by chitosan functionalization. As a result, the materials obtained differed in nitrogen content as well as in the number of total surface functional groups. The impact of the modification on the physicochemical properties of carbon carriers and their interaction with losartan potassium during adsorption and release processes was examined. The materials were characterized by various morphologies, specific surface areas (101–1180 m^2^ g^−1^), and the amount of acidic/basic oxygen-containing functional groups (1.26–4.27 mmol g^−1^). These features, along with pore sizes and volumes, had a key effect on the sorption capacity of carbon carriers towards losartan potassium (59–161 mg g^−1^). Moreover, they contributed to the differential release of the drug (18.56–90.46%). Losartan potassium adsorption onto the surface of carbonaceous materials was mainly based on the formation of hydrogen bonds and π–π interactions and followed the Langmuir type isotherm. It has been shown that the choice of the method of carbon carriers’ synthesis and their modification allows for the precise control of the kinetics of the losartan potassium release from their surface, resulting in rapid or sustained drug liberation.

## 1. Introduction

Aiming for improved pharmacotherapy, scientists progressively try to expand new mechanisms of action of active pharmaceutical ingredients (APIs), which have been known about and applied for many years. One such API example is highly tolerable losartan potassium (LOS), approved by the Food and Drug Administration (FDA) in 1995 and most commonly used for hypertension and renal disease [1]. It is also administered to prevent and control chronic fibrotic diseases such as asthma and myocardial hypertrophy, all during the reduction of platelet aggregation, decreasing the risk of thrombosis and carcinoma [2]. In recent years, it has been discovered that LOS is an angiotensin II antagonist partially capable of protecting lungs against damage caused by coronaviruses, including SARS-CoV-2 [3]. Moreover, the intranasal treatment with LOS on rodents revealed its new neuroprotective and memory-improving abilities signifying high potential in Alzheimer’s disease prevention [4,5].

The differentiated potential of LOS as a drug prompts the search for novel delivery systems that can contribute to its stabilization and a specific release mode. Up to now, only several vehicles have been proposed for LOS, such as polymer-based floating tablets [6], cellulose derivatives [7,8], self-emulsifying systems with lipids, surfactants, and oils [9,10], or patches with microneedles for transdermal delivery [11]. However, the drug carriers must be characterized by biodegradability and biocompatibility, as well as exhibit specific flexibility during preparation, which will allow their parameters to be tailored towards the desired interactions with API. When potential drug–carrier systems are presented, there is often a lack of precision in determining the maximum sorption capacity towards an active substance, as well as the influence of physicochemical properties of carriers on the ability to adsorb and then release the drug. Due to these aspects, ordered mesoporous materials, such as silica and carbons, with well-defined properties can be delivery platforms for the increasingly more complex and biologically active compounds [12,13]. Mesoporous silica is recognized for its biomedical applications [14,15], whereas the carbon materials are usually perceived as common adsorbents rather than drug-delivery systems. For instance, de Andrade et al. [16] applied micro-grain-activated carbon to compare its sorption capacity with organoclay in LOS adsorption processes. Another example is LOS removal presented by Wang and co-workers [17]. They reported a porous carbon for LOS adsorption, followed by oxidative degradation in the presence of potassium peroxymonosulfate. To the best of our knowledge, there are no reports about using porous carbons as LOS delivery platforms. The development of new and upgraded preparation techniques and functionalization methods has led to carbons’ more frequent use in drug delivery, particularly ordered mesoporous carbons (OMCs) [18,19,20,21].

OMCs are most recognized by their 2–50 nm pore size range and pore ordering, which they owe to the hard or soft templates participating during carbon syntheses. Mesoporous silica constitutes a solid matrix in the hard-templating method, whilst surfactants are used in the soft-templating approach. In the hard-template method, there are four main steps: the preparation of silica matrix, the impregnation of a matrix with precursors rich in carbon (mostly via wet impregnation or chemical vapor deposition), the carbonization of organic-inorganic (carbon-silica) composite to obtain graphite-like carbon, and the removal of silica by using hydrofluoric acid or alkalis solutions. OMCs obtained in this route are a reversed replica of the matrix with maintained ordering and high specific surface area [22,23]. Moreover, the high stability of carbon after nanocasting provides a good foundation for further carbon functionalization with a low risk of degradation of the ordered structure [24,25]. In the soft-template method, polymer surfactants such as triblock copolymers are utilized due to their ability to co-assemble with phenolic resins, which constitute carbon precursors [26,27]. In this route, the main advantage is the convenience of carrying the entire procedure in a single bottle where, under appropriate pH and temperature conditions, the reactants form a complex polymeric system. Moreover, at this stage, one can introduce carbon sources enriched in heteroatoms, or catalysts facilitating polymerization, as well as other structuring agents, which, reacting with the surfactants, may contribute to a change of the final order of the carbon material. Additionally, after carbonization, there is no need for template removal, as it completely decomposes at high pyrolytic temperatures. Fewer stages of synthesis make it relatively more profitable in terms of time and cost. However, due to the complexity of the reaction system and lack of a rigid template, polymerization may sometimes be disrupted, resulting in lower carbon surface areas obtained by this approach [28].

Depending on the method, type of template, precursors, and conditions of carbonization, the OMCs features such as structure ordering, pores size, particles morphology, and uniformity can be adjusted. As a result, it is possible to obtain porous carbon materials with high hydrothermal stability. Considering them for biomedical applications, OMCs have good biocompatibility and high purity due to the synthetic precursors [29,30,31]. Nanoporous carbons have unique structures and properties, and they might be used as diagnostic probes, nanocarriers, and biomarkers [21,32,33]. In general, nanocarbon materials were reported to have low toxicity and high biocompatibility and be non-pathogenic [34,35]. The toxicity of these materials depends on their unique physical and chemical properties. The most critical parameters are nanoparticle concentration, surface modification, and degree of aggregation in vivo. Before being administered as drug carriers, the nanoparticle biocompatibility and toxicity should be assessed. Moreover, there are increasingly more reports confirming their sustained API release, high sorption capacities, simple surface functionalization, and distinctive supramolecular π-π stacking with aromatic-drug molecules [35,36]. An interesting example of OMCs synthesis and modification specifically for tumor therapy in acidic microenvironments was presented by Huang et al. [37]. They synthesized gated channel-interconnected OMC doped with ZnO quantum dots for stimuli-responsive mitoxantrone release. The material was able to release the drug in a controlled manner upon decreasing the pH of the environment, which matches the acidity of the cancer cells. The surface modification can be also combined with heteroatom doping as in PEG-PEI-modified and nitrogen-doped mesoporous carbon for NIR light-triggered gemcitabine release [38]. The carbon was able to convert light into heat, which melts the polymeric modifier providing the release of the drug. These examples demonstrate the uniqueness and high application potential of porous carbons as carriers for drugs. Therefore, their further development is worthy of consideration.

The aim of our study was to prepare ordered mesoporous carbons using two separate synthetic methods allowing the introduction of nitrogen into one part of the materials, and a significant increase in the presence of oxygen functional groups for the second group of carriers. The main idea was to compare mesoporous carbons obtained by conventional but partially extended or modified methods, which can lead to the same structure ordering of OMCs but different textural parameters and surface chemistry. The compilation of the carbons is intended to show how the relationship with the active substance can be influenced by different carbon synthetic routes and to help in the selection of the right method for the desired adsorption/release effect. To provide such variety, 3-aminophenol (AP) was used to change N-content in carbon structure, whilst chitosan (CH) cationic biopolymer with low- and high molecular weights (MW) was applied to modify carbon surface. Chitosan is widely used in drug-delivery systems because it contributes to increasing API bioavailability and, in some cases, to improving drug retention, biodistribution, and permeability through biological membranes [39]. Subsequently, the OMCs prepared were thoroughly characterized to determine structure ordering, textural parameters, and morphology. Next, carbons with varying nitrogen content and surface-oxygen group functionalities were used as unique carriers for losartan potassium. This was accomplished by the adsorption processes of the drug, which was then released in an environment mimicking gastric fluid. The physicochemical parameters of the obtained materials were correlated with the drug adsorption and release abilities. It was shown that different nitrogen contents, as well as oxygen-containing functional groups, affect the release mode of losartan.

## 2. Experimental Section

### 2.1. Synthesis of Ordered Mesoporous Carbons with the Use of 3-Aminophenol

In order to introduce a low and high amount of nitrogen into carbon materials, the soft-templating method was carried out corresponding to Liu et al. procedure [40]. Firstly, resorcinol (POCH, 99.0%) and 3-aminophenol (Aldrich, Steinheim, Germany, 98.0%) in two various molar ratios 0.0636:0.0275 and 0.0275:0.0636, respectively, were dispersed in 540 mL of distilled water. Further sample denotation is based on the 3-aminophenol (AP) amount used, for lower-C_AP-L_ (0.0275 mol), for higher-C_AP-H_ (0.0636 mol). To the vigorously stirred aqueous solution, 0.0499 mol of hexamethylenetetramine (HMT, Aldrich, 99.0%), 0.02 mol of l-arginine (Aldrich, ≥98.0%), and 22.0 g of Pluronic F127 (Aldrich) were added. The mixture was mixed at RT for 1 h until its colour changed to yellow. Then, the bottle was closed and placed in the water bath at 70 °C for 24 h with ongoing mixing. The obtained red-brown sediment was centrifuged, washed three times with distilled water, and left at 70 °C for 12 h. Dried materials were carbonized in argon (flow rate 100 mL min^−1^), firstly, at 350 °C for 2 h, and then at 700 °C for 3 h. After the process, carbon samples were ground into fine powders.

### 2.2. Synthesis of Chitosan-Functionalized Ordered Mesoporous Carbons

Chitosan-functionalized ordered mesoporous carbons were obtained in four steps. Firstly, silica template SBA-15 was synthesized. In the bottle containing 19 mL of HCl (1.6 mol L^−1^, Avantor Performance Materials Poland S.A.), 0.5 g of Pluronic P123 (Aldrich) was dissolved at 35 °C. To the acidic solution, 1.1 mL of TEOS (tetraethyl orthosilicate, 98% wt., Aldrich) was added in a dropwise manner with continuous stirring while maintaining the previous temperature for 6 h. The mixture was closed in a polypropylene bottle and placed in an oven, at first for 24 h at 35 °C, then for 6 h at 100 °C. The resulting precipitate was separated, washed with water, and left for 12 h at 100 °C. In order to remove the template, dried material was calcined for 8 h at 550 °C.

Subsequently, a carbon impregnating solution was prepared via dissolving 1.25 g of sucrose (Aldrich) in 0.14 mL of H_2_SO_4_ (Avantor Performance Materials) and 5 mL of water. Then, the silica SBA-15 template was impregnated twice with as-prepared sucrose solution and heated in two steps—at 100 °C for 6 h and at 160 °C for 6 h. The silica-carbon composites were additionally subjected to the sucrose solution but less concentrated, prepared from 0.8 g of sucrose, 0.09 mL of H_2_SO_4_, and 5 mL of water. The impregnated material was heated in the same as previous manner, at 100 °C for 6 h and 160 °C for 6 h, followed by carbonization at 900 °C for 3 h (heating rate 2.5 °C min^−1^) in an argon atmosphere. To remove the remaining SBA-15 matrix, the material was washed twice with 200 mL of 5% HF solution (Avantor Performance Materials), then with water and ethanol. Next, the carbon material was placed in the oven at 100 °C overnight and labeled as C_SBA-15_.

In order to modify C_SBA-15_ with chitosan, the sample surface was firstly oxidized with 1 mol L^−1^ of ammonium persulfate solution (APS, Aldrich), and 5 g of C_SBA-15_ and 30 mL of APS solution were magnetically mixed and heated at 60 °C for 6 h under reflux. Then, the material was separated, washed with water, and dried at 100 °C overnight.

After introducing oxygen functional groups to the carbon surface, chitosan was grafted onto oxidized C_SBA-15_. In two different bottles, the solutions of chitosan with low (CH-L) and high (CH-H) molecular weight were prepared as follows: 0.5 g of each chitosan was dispersed in 50 mL of NaCl solution (0.125 mol L^−1^) and 50 mL of acetic acid solution (0.1 mol L^−1^). After chitosan dispersion, 2.5 g of oxidized C_SBA-15_ was added to each solution, and mixtures were stirred at RT for 8 h. Afterwards, materials were separated, washed with water, and dried at 100 °C for 12 h. The obtained chitosan-modified carbon materials were denoted accordingly to the modifier molecular weight—C_CH-L_, and C_CH-H_.

### 2.3. Characterization of Materials

#### 2.3.1. Powder X-ray Diffraction

To establish structure ordering of mesoporous carbon carriers, their low-angle XRD profiles were made by D8 Advance Diffractometer (Bruker, Cu Kα1 radiation λ = 1.5406 Å) with step size 0.02°.

#### 2.3.2. Transmission Electron Microscopy

To provide an overview of the pore ordering, TEM images were registered. Carbon-powdered samples were embedded on a grid with a perforated carbon film and placed into JEOL 2000 electron microscope operating at 80 kV.

#### 2.3.3. Elemental Analysis

The CHNS/O element content was measured by using Thermo Scientific FLASH 2000 Elemental Analyzer (OEA).

#### 2.3.4. Scanning Electron Microscopy

The morphology of carbon materials was determined by scanning electron microscope (SEM, FEI Helios NanoLab 660). The conditions of the analysis were as follows: room temperature, high-vacuum mode of 7 × 10^−4^ Pa, and modes were field-free (FF) and high-resolution (HR) with 10 kV acceleration voltage and 0.2 nA beam current. The resulting images were registered by secondary electrons using the Everhart-Thornley Detector for FF and Through-Lens Detector for HR.

#### 2.3.5. Low-Temperature Nitrogen Sorption

The textural parameters of the synthesized carbon samples were defined by low-temperature nitrogen adsorption/desorption isotherms, which were obtained after conducting the process at −196 °C with the use of a Quantachrome Autosorb IQ apparatus. Before the measurement, in order to remove moisture, materials were degassed for 3 h at the following temperatures: C_AP-L_ and C_AP-H_ at 200 °C, C_SBA-15_ at 300 °C, and C_CH-L_ and C_CH-H_ at 100 °C, to avoid the decomposition of chitosan. The specific surface area was ascertained via calculations using Brunauer–Emmett–Teller (BET) method, while the average pore size was evaluated using Barret–Joyner–Halenda (BJH) method.

#### 2.3.6. Surface Oxygen Functional Groups

The Boehm titration method was applied to establish the amount of acidic and basic oxygen functional groups on the surface of carbon materials. Carbon samples required preparation before titration as follows: 0.1 g of carbon was immersed in 10 mL of NaOH solution (0.1 mol L^−1^, POCH) to neutralize acidic groups. The mixture was agitated for 24 h at RT, then the material was separated and the solution was titrated with HCl solution (0.1 mol L^−1^, Chempur) to determine acidic groups amount. In turn, to calculate basic functional groups amount, the same amount of carbon was dispersed in HCl solution (0.1 mol L^−1^). Then, the mixture was agitated and separated similarly as previously, followed by titration with NaOH solution (0.1 mol L^−1^). All titration procedures were conducted in the presence of methyl orange as an indicator.

#### 2.3.7. Infrared Spectroscopy

Fourier-transform infrared spectra (FT-IR) were registered by using FT-IR Bruker IFS 66v/S 161 spectrometer. The carbon samples and carbon-losartan systems (ca. 0.3 mg) were grounded with anhydrous KBr (ca. 0.25 mg), followed by tablets formation. The analysis was conducted in a wavenumber range of 4000–400 cm^−1^ (resolution of 0.5 cm^−1^; the number of scans: 64).

### 2.4. Losartan Potassium Adsorption Studies

The obtained carbon materials were applied as vehicles for losartan potassium, therefore adsorption abilities of materials towards drug have been measured via Agilent Cary 60 UV-Vis spectrophotometer at a wavelength of 233 nm. The specific amount of each material (25 mg) was dispersed in losartan solutions of concentration range 5–100 mg L^−1^ and agitated at RT for 24 h. Subsequently, carbon carriers with adsorbed losartan were separated, and the absorbance of the remaining solutions was measured, followed by calculations of sorption capacity using the formula below.

(1) $$q_{e}=\frac{(C_{0}-C_{e})\cdot V}{m}$$

*q_e_*—the amount of adsorbed losartan onto nanoporous carbon carriers (mg g^−1^),
*C*_0_—the initial concentration of losartan (mg L^−1^),
*C_e_*—the equilibrium concentration of losartan after adsorption (mg L^−1^),
*V*—the volume of losartan solution (L),
*m*—the mass of carbon adsorbent (g).

The obtained adsorption results were analyzed and fitted to two types of adsorption isotherms of the Langmuir and Freundlich [41,42]. By referring to the correlation coefficients (R^2^) of the models, the mechanism of adsorption has been established.

The Langmuir isotherm is described by the following equation:

(2) $$\frac{C_{e}}{q_{e}}=\frac{1}{q_{m}K_{L}}+\frac{C_{e}}{q_{m}}$$

*q_m_*—the maximum monolayer adsorption capacity of carbon adsorbent (mg g^−1^),
*K_L_*—the Langmuir constant denoted the energy of adsorption and affinity of binding sites (L mg^−1^).

The Freundlich isotherm and its linear form are described by the Equation (3):

(3) $$\ln q_{e}=\ln K_{f}+\frac{1}{n}\ln C_{e}$$

*K_F_*—Freundlich constant, which relates to the adsorption capacity of the adsorbent (mg g^−1^ (L mg^−1^)^1/n^),
*n*—constant, which indicates how favorable is the adsorption process.

### 2.5. Losartan Release Studies

The release rates of losartan potassium from carbon carriers were tested in a paddle apparatus (Agilent 708-DS; Agilent, Santa Clara, CA, USA) with a rotation speed of 50 rpm. The carrier-API systems were weighted to gelatin capsules and placed into springs acting as sinkers preventing flotation in the vessel. The test was conducted in the HCl solution (0.1 mol L^−1^), according to FDA recommendations. The temperature of the medium was 37 °C ± 0.5 °C. During the study, at specified time points, 5 mL of samples was collected halfway between the surface of the medium and the paddle top edge, no closer than 1 cm from the wall of the vessel, and refilled by a clean medium of the temperature of 37 °C ± 0.5 °C. The vessels were covered during the test, and the study medium temperature was maintained. The analysis of the samples was performed by the HPLC method (Shimadzu, Tokyo, Japan) at a wavelength of 233 nm. The column (Phenomenex 250 mm × 4.6 mm; 5 µm; Torrance, CA, USA) oven was set at 30 °C; the stationary phase was 0.1% formic acid and acetonitrile (40:60) at the flow of 0.8 mL min^−1^.

To establish the type of losartan liberation, datasets were correlated to five mathematical models: zero-order (% LOS release vs. time) (4), first-order (log of % LOS release vs. time) (5), Higuchi’s model (% LOS release vs. square root of time) (6), Hixson–Crowell (cube root of % LOS remaining vs. time) (7), and Korsmeyer–Peppas model (log of % LOS vs. log time) (8) [43]. The criterion of fitting was the correlation coefficient (R^2^), whose highest value indicated the most probable release mechanism.

(4) $$F_{t}=k_{0}t_{e}$$

(5) $$F_{t}=1-e^{-kt}$$

(6) $$F_{t}=k_{H}\sqrt{t}$$

(7) $$\sqrt[{3}]{F_{0}}-\sqrt[{3}]{F_{t}}=k_{HC}t$$

(8) $$F_{t}=kt^{n}$$

*F_t_*—the fraction of losartan released in time,
*F*_0_—the initial amount of losartan in the nanocarrier,
*k*_0_, *k_t_*, *k_H_*, *k_HC_*, *k*—the release constants of particular kinetic models,
*n*—the diffusion exponent.

### 2.6. Permeability Study

Gastrointestinal (GIT) permeability was performed using PAMPA (parallel artificial membrane permeability assay) based on passive diffusion. The samples were dissolved in DMSO, mixed with donor solution (pH 1.2), then they were added to donor compartments on a 96-well plate. The system consists also of another 96-well plate with acceptor chambers. Donor and acceptor compartments are separated by a 120 μm thick microfilter disc coated with a 20% (*w*/*v*) dodecane solution of a lecithin mixture (Pion, Inc., Billerica, MA, USA). Both plates were combined as one and incubated for 3 h at the temperature of 37 °C in a humidity-saturated atmosphere. After the time of incubation, the compartments were split and their content was analyzed using the HPLC method (see Section 2.5). The apparent permeability coefficient (Papp) was calculated using the following equation:

(9) $$P_{app}=\frac{-ln\left(1-\frac{C_{A}}{C_{equilibrium}}\right)}{S\times \left(\frac{1}{V_{D}}+\frac{1}{V_{A}}\right)\times t}$$

(10) $$C_{\mathrm{equilibrium}}=\frac{C_{D}\times V_{D}+C_{A}\times V_{A}}{V_{D}+V_{A}}$$

where:

*V_D_*—donor volume,
*V_A_*—acceptor volume,
*C_equilibrium_*—equilibrium concentration,
*S*—membrane area,
*t*—incubation time (in seconds).

## 3. Results and Discussion

### 3.1. Physicochemical Characterization of Nanoporous Carbon Carriers

**Figure 1:**
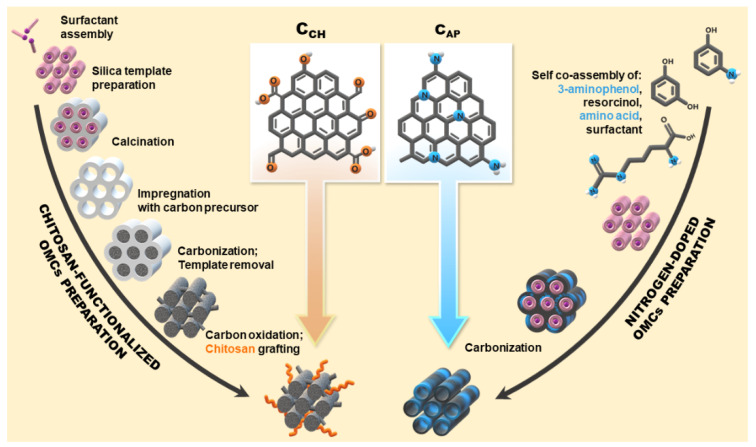
Scheme representing syntheses of carbon carriers via different routes. On the left: chitosan-modified ordered mesoporous carbons (C_CH_) via the hard-templating method, followed by oxidation and chitosan grafting. On the right: nitrogen-doped ordered mesoporous carbons (C_AP_) through soft-templating method with 3-aminophenol and amino acid (l-arginine) as N-dopants.

The divergent synthetic routes for carbon carrier preparation, i.e., soft-templating method with the use of 3-aminophenol (AP) and nanocasting followed by chitosan (CH) grafting, were utilized to generate nitrogen and oxygen-containing functional groups in their structures (Figure 1). Nitrogen-enriched mesoporous carbons with low (C_AP-L_) and high (C_AP_-_H_) amounts of AP were compared to the carbons functionalized with low (C_CH-L_) and high (C_CH-H_) molecular weight chitosan. The motivation for such modifications of mesoporous carbons was to establish how the functional groups will interact with losartan potassium and the assessment of their application potential as carriers in the processes of drug adsorption and release.

**Figure 2:**
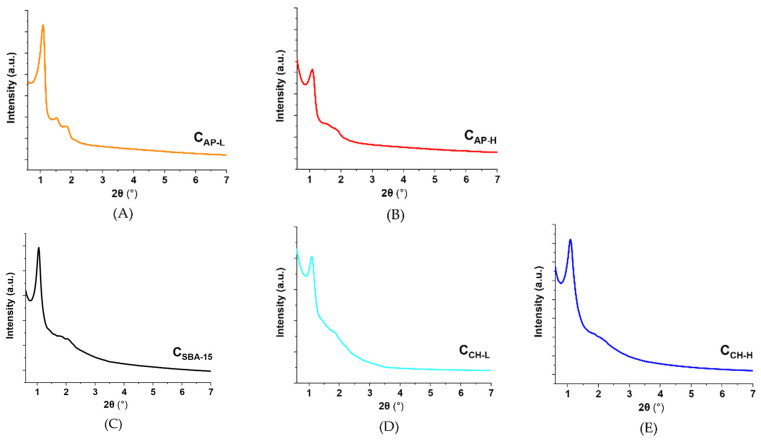
XRD profiles in the small-angle range of the carbon materials: C_AP-L_ (**A**), C_AP-H_ (**B**), C_SBA-15_ (**C**), C_CH-L_ (**D**), and C_CH-H_ (**E**).

The carbon materials obtained via two different approaches revealed ordered structures as evidenced by the peaks visible in the small-angle XRD profiles (Figure 2). The characteristic sharp peak at 2θ = 1° observed for all samples corresponds to the (211) plane and suggests the presence of hexagonal pore arrays. The differences in the intensity of this peak are especially noticeable for the C_AP-L_ and C_AP-H_ materials. In the case of the first sample, the reflection is more pronounced. It can be concluded that using a lower amount of AP provides better mesoporous structure ordering. Moreover, other peaks in the range between 1.5 and 2.0° are also sharper in the XRD profile of the C_AP-L_ sample. The diffractograms of carbon materials based on C_SBA-15_ contain less intensive reflections in the analyzed range, which indicate less ordered hexagonal structure affiliated to the p6mm space group. It was established that the use of chitosan for functionalization of C_SBA-15_ does not significantly affect the ordering of the mesoporous structure. Only in the case of C_CH-L_ material, a slight reduction in peaks intensity is noticed. It implies that smaller chitosan molecules could attach not only to the carbon surface but also could be partially loaded into the mesopores [44].

**Figure 3:**
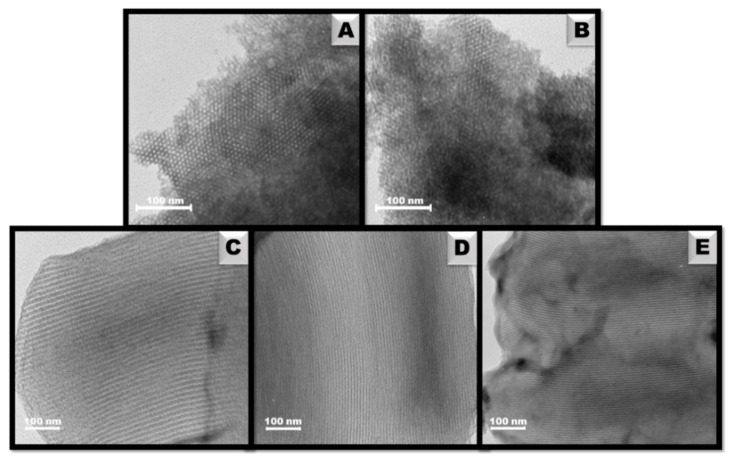
TEM images of ordered mesoporous carbons: C_AP-L_ (**A**), C_AP-H_ (**B**) cross-section revealing hexagonal honeycomb order; C_SBA-15_ (**C**), C_CH-L_ (**D**), and C_CH-H_ (**E**) side view showing independent cylindrical channels.

The arrangement of the pore structure was further confirmed by TEM images of OMCs (Figure 3). Referring to the XRD results, all materials showed a hexagonal ordering, which corresponds to the earlier reports [45,46]. This is particularly evident for the carbons obtained by the soft-templating method, which are shown in Figure 3A,B. The images present a detailed cross-section of the porous network, from which it is clear that the pores are hexagonally arranged resembling a honeycomb structure. TEM images of carbons obtained by the hard-template method are displayed in Figure 3C–E. They demonstrate the individual pore channels with a high degree of order. Interestingly, pristine carbon, as well as functionalized materials, are characterized by the same pore ordering. Thus, there is a lack of the negative effect of chitosan modification on material structures proving their stability.

The analysis of elemental composition confirmed successful enriching of carbon materials with heteroatoms, i.e., nitrogen in the case of C_AP-L_ and C_AP-H_ and oxygen for C_CH-L_ and C_CH-H_ (Table 1). Using AP, HMT, and l-arginine during the carbon synthesis led to the introduction of 3.10 wt.% and 6.42 wt.% of nitrogen for C_AP-L_ and C_AP-H_, respectively. The higher amount of aminophenol added to the synthetic mixture increased percentage content of nitrogen in carbon structure, which signifies that this method can be further adjusted for the desired N-doping. The carbon materials obtained by the soft template method (C_AP-L_, C_AP-H_) exhibit a more carbon-rich structure (ca. >86 wt.%) compared to samples synthesized via hard-templating approach (C_SBA-15_, C_CH-L_, C_CH-H_). This is due to the fact that aminophenol, besides doping samples with nitrogen, also constitutes a source of carbon together with resorcinol. Nano-casted carbons contain about 69–82 wt.% of carbon but significantly more oxygen. C_SBA-15_ exhibits 14.84 wt.% of oxygen in the structure, whilst CH-modified materials display 14 wt.% more. The oxygen quantity is 28.83 and 29.02 wt.% for C_CH-L_ and C_CH-H_ samples, respectively. Chitosan is a polymer rich in oxygen functionalities; therefore, it increased the amount of oxygen in the carbon structure. However, it should be highlighted that before modification of C_SBA-15_ with chitosan, carbon material was oxidized to generate oxygen moieties. They facilitated further modification with polymer but also partially increased oxygen content [18].

**Figure 4:**
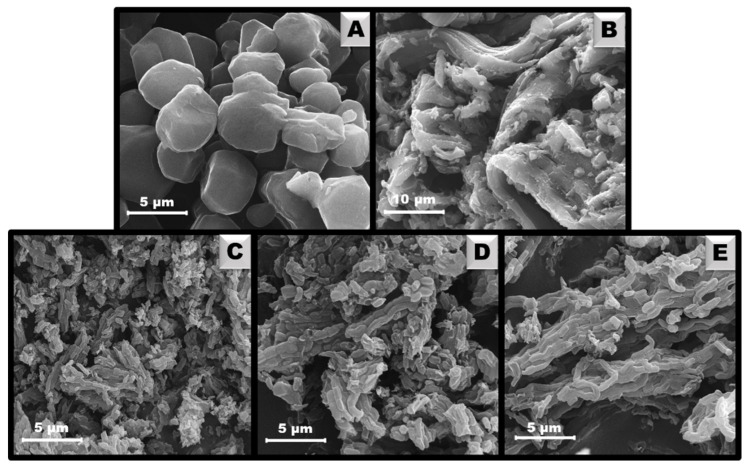
SEM images of ordered mesoporous carbons: C_AP-L_ (**A**), C_AP-H_ (**B**), C_SBA-15_ (**C**), C_CH-L_ (**D**), and C_CH-H_ (**E**).

The topography of the surface and shape of carbon particles were investigated by scanning electron microscopy and SEM images are depicted in Figure 4. Among all materials, C_AP-L_ is characterized by the most unique polyhedron-like particles. Notably, it is possible to separate single particles from others and estimate their size, which is ~5 µm (Figure 4A). Such morphology corresponds to the most pronounced XRD peaks for the maintained p6mm mesostructure of C_AP-L_. Using a higher amount of aminophenol during carbon synthesis led to the loss of the uniform polyhedrons, and wavy heterogeneous folds were formed in C_AP-H_ (Figure 4B). Li et al. [40] reported that a high amount of 3-aminophenol may cause a partial degradation of carbon structural ordering, thus it disrupts the creation of uniform particles. The materials synthesized by the hard template method utilizing SBA-15 as a template (Figure 4C–E) are very similar to each other, and the worm-like particles of carbons resemble the shape of the applied silica [47]. Elongated particles of sizes 1–3 µm also appear in functionalized samples C_CH-L_ and C_CH-H_. Hence, it can be concluded that neither oxidation nor modification with chitosan affected the morphology of the carbon material.

The textural parameters of carbon samples are listed in Table 2. They were established via proper calculations based on low-temperature adsorption/desorption of nitrogen. The carbon materials prepared by the soft-templating method have the smallest surface area: C_AP-L_—107 m^2^ g^−1^ and C_AP-H_—101 m^2^ g^−1^. The reason for such values is possible disruption occurrence, during micelle assembly and polymerization, caused by adding nitrogen-rich reagents to synthetic mixture. C_AP-L_ stands out from other materials with the highest pore diameter of 9.22 nm, which has lowered almost 5-fold for C_AP-H_ resulting in 1.67 nm. In contrast, C_SBA-15_ revealed the most developed surface area of 1180 m^2^ g^−1^ decreasing after modification with chitosan to 547 m^2^ g^−1^ and 483 m^2^ g^−1^ for C_CH-L_ and C_CH-H_, respectively. Firstly, carbon surface oxidation and newly generated functional groups led to a surface area reduction. It is also visible by the lowered pore volumes and diameters from 1.17 cm^3^ g^−1^ and 4.23 nm for pristine carbon, to 0.47/0.42 cm^3^ g^−1^ and 3.42/3.29 nm for the modified samples. Additionally, attaching the chitosan onto the carbon surface could partially block access to the pores. Lastly, the reduced surface area of the modified carbons can be also explained by the lack of porosity in the polymer itself.

**Figure 5:**
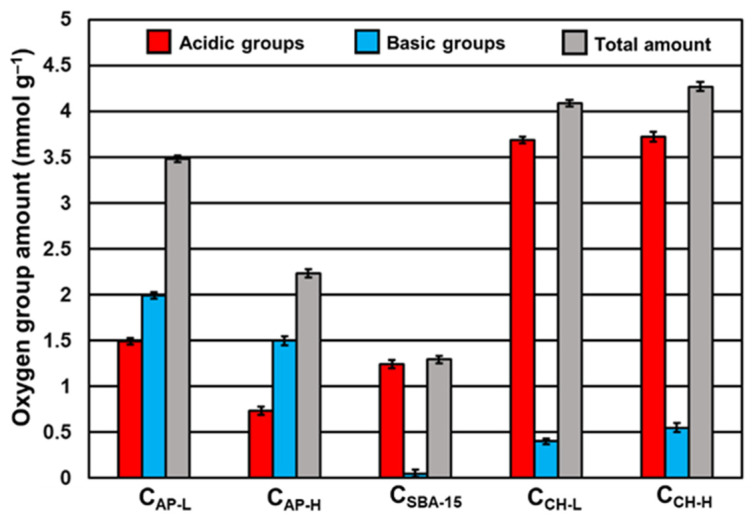
Amount of acidic and basic oxygen functional groups on the carbon carriers’ surface determined via Boehm method.

Before analyzing the adsorption/release abilities of nanoporous carbon carriers, their surface chemistry has to be determined. Oxygen-containing functional groups have a major influence on both sorption capacities towards drug and the manner of its liberation from the delivery platform. The amount of acidic and basic moieties present on the surface of mesoporous carbons established via the Boehm method is displayed in Figure 5 [48]. All materials revealed different contents of oxygen-containing functional groups. Chitosan-functionalized carbons exhibit the richest surface chemistry (total number of groups over 4 mmol g^−1^), whilst N-doped materials have the largest number of basic groups. Interestingly, C_AP-L_ has more basic functional groups (1.99 mmol g^−1^) than C_AP-H_ (1.50 mmol g^−1^). Nitrogen introduction could lead to the generation of amide groups, while increased basicity is also influenced by groups such as quinones, chromenes, and pyrenes [49,50]. Thus, there is no dependence that higher quantities of nitrogen cause higher basicity of carbon surface. The use of the smaller amount of aminophenol and more resorcinol in the synthesis of C_AP-L_ ultimately resulted in the total number of acidic and basic groups of 3.48 mmol g^−1^, where for C_AP-H_ it is 2.23 mmol g^−1^. The surface of non-modified C_SBA-15_ shows acidic nature. The number of oxygen functional groups was found to be 1.24 mmol g^−1^. The oxidation process and subsequent modification of the C_SBA-15_ surface with chitosan resulted in a significant increase in the total number of functional groups, with a predominance of acidic ones. Both chitosan-modified carbons, C_CH-L_ and C_CH-H,_ have a comparable number of acidic groups, 3.68 mmol g^−1^ and 3.72 mmol g^−1^, respectively. It suggests that the molecular weight of chitosan has little impact on the number of oxygen moieties.

### 3.2. Adsorption and Release of Losartan Potassium

**Figure 6:**
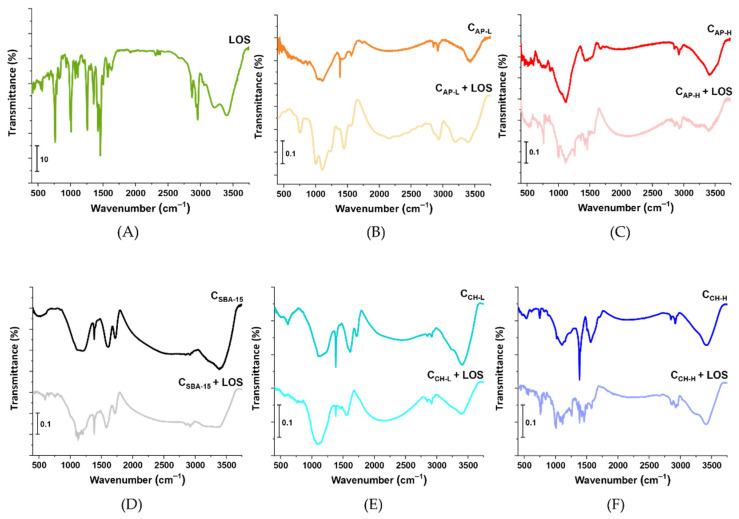
FT-IR spectra of losartan potassium (**A**), pure carbon materials and carrier-API systems: C_AP-L_ (**B**), C_AP-H_ (**C**), C_SBA-15_ (**D**), C_CH-L_ (**E**), and C_CH-H_ (**F**).

Nanoporous carbon materials were used as delivery platforms for losartan potassium. To establish how losartan binds to the carriers, the FT-IR spectra of each carbon material and carrier–drug systems are demonstrated in Figure 6 and further analyzed. In the FT-IR spectrum of losartan potassium, a band at 671 cm^−1^ characteristic for -OH out-of-plane bending vibrations is visible (Figure 6A) [51]. The band at 761 cm^−1^ can be associated with stretching vibrations of C–Cl. At the wavenumber of 788 cm^−1^, the band assigned to aromatic C–H out-of-plane bending vibrations is identified. C–O stretching vibrations and breathing of aromatic rings were recorded in the region 997 to 1074 cm^−1^. Due to the wagging vibrations of C–H in the aliphatic chain and stretching vibrations of C–N, a band at 1257 cm^−1^ appeared in the FT-IR spectrum. In addition, the absorption bands at 1423 cm^−1^ and 1458 cm^−1^ are related to methyl C–H symmetric bending vibrations and –CH_2_– and C–H bending vibrations in the aromatic rings. The band at the wavenumber of 1579 cm^−1^ is ascribed to N=N and C=C stretching vibrations. The bands at 2870 cm^−1^ and 2931 cm^−1^ prove the presence of symmetric stretching vibrations of –CH_2_–. In turn, the band at 2958 cm^−1^ is associated with asymmetric –CH_3_ vibrations.

The FT-IR spectra of all mesoporous carbon materials contain wide bands at ~3400 cm^−1^ appearing from several phenomena: the O–H stretching vibrations of the adsorbed water, the vibrations of –COOH, and the intramolecular hydrogen bonds [52,53]. Moreover, every sample after losartan adsorption indicates in this region a new band at 3250 cm^−1^ coming from the new N–H bond, which is one of the confirmations of the presence of the drug on the carbon surfaces. The repeating in all cases bands at ~2850 cm^−1^ and ~2920 cm^−1^ correspond to aliphatic –CH_3_ and/or –CH_2_– groups. By analyzing FT-IR spectra of the carbon carriers individually, it can be stated that C_AP-L_ and C_AP-H_ indicated broad bands in the range 870–1340 cm^−1,^ which can be assigned to the N–H bonds and their out-of-plane deformation. The sharp band at 1392 cm^−1^ visible in the spectrum of the C_AP-L_ sample may belong to the –CH_3_ symmetrical bending vibrations and the N–CH stretching vibrations [54]. There is no such sharp band in C_AP-H_ spectra. However, the broad band in the region of 1357–1615 cm^−1^ can be assigned to overlapping vibrations derived from either surface –OH groups or C=C–H, C–N– in heterocyclic rings, and carboxyl-carbonates structures [55]. A new band emerged in the spectra of C_AP-L_+LOS and C_AP-H_+LOS at ~765 cm^−1^, which is a slightly shifted band from stretching vibrations of C–Cl present in the losartan. The sharp bands at ~1600 cm^−1^ ascribed to C=C and C–H from the aromatic ring but also at ~1725 cm^−1^ typically assigned to the stretching vibrations from C=O are observed in the spectra of pristine C_SBA-15_ and C_CH-L_ samples. Non-modified and chitosan-modified materials revealed a wide band at 850–1320 cm^−1^ corresponding to the N-H bonds similar to N-doped carbons. These materials are also rich in carboxylic, ester, etheric, and phenolic groups. Moreover, new bands appeared after API adsorption, most notably at 995 cm^−1^, 1115 cm^−1^, and 1265 cm^−1,^ which can be assigned to the stretching vibrations of C–O, C–N, and wagging vibrations of C–H from losartan.

In conclusion, the FT-IR spectra of all functionalized mesoporous carbons after adsorption of losartan exhibit bands characteristic for the selected drug. However, it is worth noting that some bands are shifted or their intensity changes, which is the result of drug binding to the surface of the mesoporous carbon carriers.

**Figure 7:**
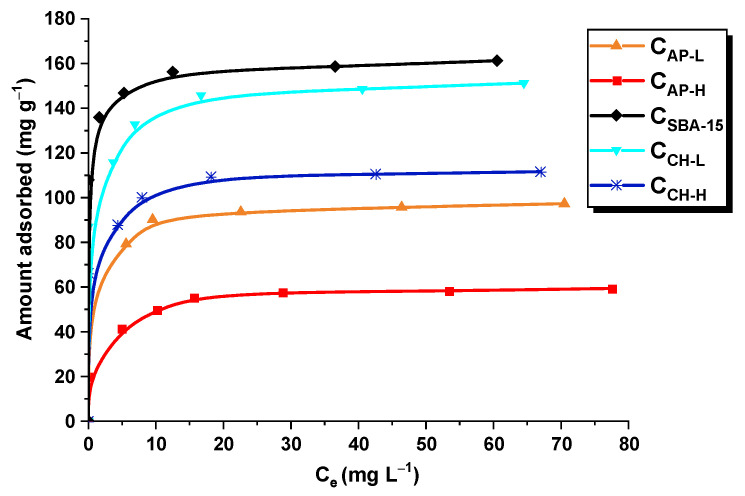
Adsorption isotherms of losartan potassium on the surface of C_AP-L_, C_AP-H_, C_SBA-15_, C_CH-L_, and C_CH-H_ carriers.

Among all synthesized nanocarriers, pure ordered mesoporous carbon C_SBA-15_ is characterized by the highest sorption capacity in relation to losartan (161 mg g^−1^), which can be attributed primarily to its most developed specific surface area and large pore volume (Figure 7). These results are similar to our previous reports comparing paracetamol adsorption on carbons obtained by hard and soft template approaches [18]. Due to the fact that losartan occurs in the anionic form at a pH 7 and the carbon surface is negatively charged, electrostatic interactions cannot take place. Therefore, drug adsorption onto the surface of C_SBA-15_ is mainly based on the formation of hydrogen bonds and π–π interactions. In the case of chitosan-modified mesoporous carbons, the sorption capacity towards losartan decreases to 151 mg g^−1^ for C_CH-L_ and 112 mg g^−1^ for C_CH-H_ compared to C_SBA-15_. It can be explained by the presence of a large number of acidic oxygen-containing moieties on the surface of these materials, which may partially repulse the functional groups of hydrophobic losartan molecules. Moreover, the modification with chitosan caused a deterioration of the textural parameters of the samples, which has been also reported for chitosan-coated silica particles a ibuprofen delivery [56]. Some of the entrances to the mesopores/micropores are blocked by chitosan functional groups, preventing the diffusion of losartan molecules into their interior. On the other hand, when analyzing carbons containing nitrogen in their structure, it should be emphasized that C_AP-L_ (97 mg g^−1^) exhibits a better sorption capacity than C_AP-H_ (59 mg g^−1^), which indicates its greater affinity for the studied drug (Figure 7). This is primarily due to the presence of basic functional groups that can electrostatically interact with the negatively charged functional groups of the drug. However, other specific/chemical interactions must be also taken into account when analyzing the mechanism of the losartan adsorption process, e.g., hydrogen bond formation, π–π interactions.

**Figure 8:**
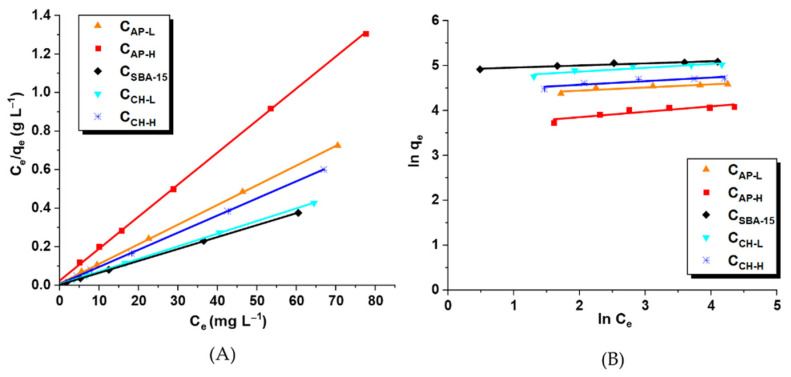
The fitting of experimental data of adsorption of losartan potassium on the surface of C_AP-L_, C_AP-H_, C_SBA-15_, C_CH-L_, and C_CH-H_ carriers to the Langmuir (**A**) and Freundlich (**B**) models.

The experimental data of losartan adsorption on the surface of C_SBA-15_, C_CH-H_, C_CH-L_, C_AP-H_, and C_AP-L_ nanocarriers at equilibrium were fitted to the linear form of Langmuir and Freundlich isotherm models (Figure 8). The adsorption parameters estimated based on these two models are listed in Table 3. Evaluating the R^2^ values for the systems studied, it was noticed that the Langmuir model described better drug adsorption on the carbon materials than the Freundlich ones. The K_L_ parameter in the Langmuir equation is related to the drug affinity to adsorbents. Comparing the obtained values of this parameter, a greater affinity of losartan to pristine and chitosan-modified ordered mesoporous carbons C_SBA-15_ was observed.

**Figure 9:**
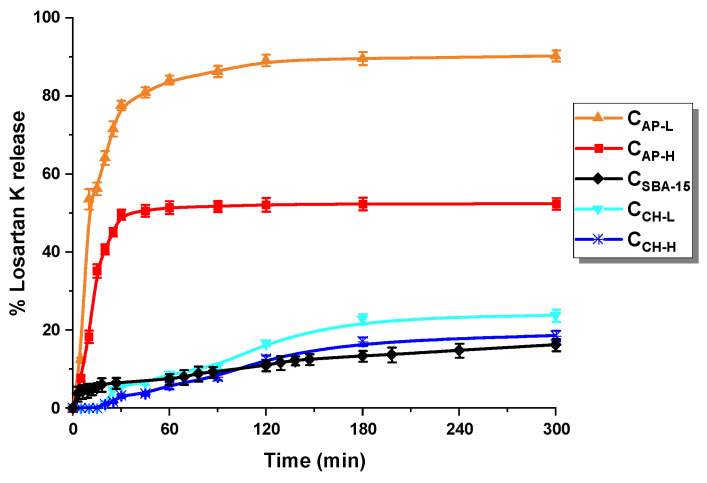
Release profiles of losartan potassium from carbon carriers: C_AP-L_, C_AP-H_, C_SBA-15_, C_CH-L_, and C_CH-H_ in acidic medium (HCl 0.1 mol L^−1^).

The losartan release results in the acceptor simulating gastric fluid (0.1 mol L^−1^ HCl) are presented in Figure 9. All carbon materials indicated the ability to release the selected drug. The percentage of losartan liberated from the nanoporous carriers ranges from ~16% to ~90% depending on the carbon platform applied. Soft-templated carbons (C_AP-L_, C_AP-H_) stand out with the rapid release of losartan potassium. C_AP-L_ sample liberated 84% of adsorbed API within 60 min, with less intense release in the following hours-up to 90.46%. On the other hand, in the case of the second nitrogen-doped carrier (C_AP-H_), the drug concentration (52.35%) in the receptor fluid stabilizes after 30 min of the process. The burst of losartan from these carriers signifies that it was adsorbed on the external surface rather than in the pores, due to their relatively low porosity. Moreover, at pH 1.2 functional groups of carbons and losartan itself (due to the acidic tetrazole ring) are protonated. Hence, electrostatic repulsion could occur leading to increased drug liberation [57,58,59]. The lack of ~48% of losartan discharge from C_AP-H_ proves its greater affinity in acidic media to carbon with less amount of surface functional groups. Additionally, despite the comparable surface area of C_AP-L_ and C_AP-H_, the second material has a lower average pore diameter and pore volumes, which intensifies the interaction between the pore walls and losartan ultimately leading to lowered total drug release.

The mesoporous carbons obtained by the hard-templating method exhibited the contrary release behavior. The pristine sample C_SBA-15_ liberated the least API (16.22%), which is dictated by the most developed porous structure. Hence, losartan trapped in carbon pores diffused slowly and in small amounts. The modification with chitosan led to slightly increased drug desorption in a more controlled manner. The functionalized materials C_CH-L_ and C_CH-H_ released 23.76% and 18.56%, respectively. The increased percentage of losartan release is due to decreased porosity of the carriers and partial drug adsorption on their external surfaces. Moreover, more oxygen functional groups prompt an increase in the efficiency of the liberation of adsorbed losartan. By taking an overall look at losartan release from soft- and hard-templated carbons, the latter appear to differ less. C_SBA-15_ and chitosan-modified carbon textural parameters are unalike when one considers S_BET_ and pore volumes, hence smaller differences in liberation must relate to disparate phenomena. In the case of non-modified carbon, LOS is mostly trapped within the carbon pore network; high affinity between adsorbent and adsorbate result in slow API liberation. However, where it comes to the modified materials, due to the modifier-induced decrease in specific surface area, the high affinity of LOS occurs due to the chitosan itself. Song et al. [60] reported a chitosan-modified carbon for pesticide (emamectin benzoate) delivery, which sustained the escape of active ingredient due to the metastable combination of the outer layer of the adsorbate. The strong interaction between chitosan and drug consequently results in similar slow desorption of the LOS; however, it opens the pathway to regulate release behavior. Nonetheless, the presence of varied results suggests that by using chitosan as a modifier with different molecular weights the total drug release can be modulated.

The in vitro losartan release data were evaluated kinetically using various mathematical models presented in Table 4. It was found that drug liberation from N-doped carbons fits the best to the first-order model: R^2^ = 0.932 and 0.974 for C_AP-L_ and C_AP-H_, respectively. Therefore, it can be stated that the release is dependent on the drug concentration, rather than on the carrier. This suggests that if more API would be adsorbed onto the carbon vehicle, the faster release would occur. The losartan liberation data for C_SBA-15_ and C_CH-H_ exhibit the highest correlation coefficients (0.988; 0.963) when the Higuchi model is applied. This model attests to a controlled manner of release and indicates that the diffusivity of the drug is constant. Additionally, it confirms that the initial losartan concentration in the system is higher than the carrier solubility in the acceptor medium. The last sample (C_CH-L_) showed the highest and most similar values of the correlation coefficient for two models, the zero-order (0.971) and the Hixson–Crowell (0.970) models. The first model indicates the sustained drug liberation from the vehicle, whilst the second model signifies the dependence between carrier surface area and API dissolution [61].

**Figure 10:**
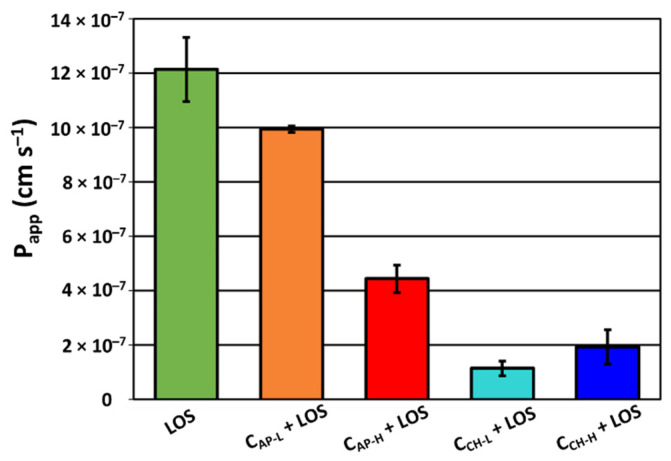
Gastrointestinal mean permeability of intrinsic losartan potassium and after liberation from functionalized ordered mesoporous carbons.

Losartan potassium belongs to class III of drugs with low solubility and high permeability according to the biopharmaceutics classification system (BCS) [62]. Its permeability coefficient was 12.14 × 10^−7^ cm s^−1,^ which corresponds to literature data for well permeable compounds (>10.00 × 10^−7^ cm s^−1^). The permeability of losartan introduced onto the N-doped and chitosan-modified carbons is depicted in Figure 10. API liberated from the carriers indicated reduced permeability in comparison to the pure losartan. The smallest alteration of Papp value was caused by the carrier C_AP-L_ (9.94 × 10^−7^ cm s^−1^). It correlates with the most efficient release of drug from this carrier. C_AP-H_ exhibited 4.44 × 10^−7^ cm s^−1^ permeability. The losartan delivery systems based on chitosan-modified carbon carriers have the lowest permeability of 1.14 × 10^−7^ cm s^−1^ (C_CH-L_), and 1.92 × 10^−7^ cm s^−1^ (C_CH-H_).

## 4. Conclusions

In this article, advanced losartan potassium delivery systems based on functionalized ordered mesoporous carbon materials of different morphology were presented. To obtain carbon materials with desired physicochemical properties, two synthetic approaches were applied. The first, the soft-templating method with Pluronic F127, was modified by adding nitrogen sources-3-aminophenol and l-arginine. Thanks to this approach, it was possible to fabricate nitrogen-doped mesoporous carbons characterized by the presence of basic functional groups and polyhedral or corrugated particles. The second group of carbonaceous carriers was synthesized by the hard-templating method, relying on the SBA-15 silica matrix, followed by chitosan biopolymer functionalization. These materials exhibit a high amount of acidic surface oxygen-containing moieties and worm-like morphology. The modification of mesoporous carbons brought about a reduction in the ordering of their hexagonal structure, and, subsequently, the textural parameters deteriorated compared to the pristine sample. The sorption capacities of carbon vehicles towards losartan potassium are dependent on their porosity and morphology, as well as amount and nature of surface functional groups. Materials synthesized by the hard-templating method and modified with chitosan adsorb more API than nitrogen-enriched carbons. This is due to their higher specific surface area and pore volumes. Moreover, a high concentration of surface functional groups favors the intensification of host–guest interactions. Among applied adsorption isotherm models, the Langmuir one shows the best fit with the experimental data. The monolayer sorption capacities of mesoporous carbon samples are in the range 59–161 mg g^−1^. It has been shown that the choice of nanocarrier synthesis method, its parameters, and its modification have a significant impact on the kinetics of losartan potassium release, which can be modulated depending on the needs. The use of nitrogen-doped carbons as a delivery platform resulted in the faster and more effective liberation of the drug. On the other hand, in the case of chitosan-functionalized nanocarriers, the release of losartan potassium was gradual and occurred in a controlled manner. However, within 5 h of the process, the lower drug concentration in the acceptor fluid was determined.

## Figures and Tables

**Table 1:** The elemental composition of carbon carriers.

Material	N (wt.%)	C (wt.%)	H (wt.%)	O (wt.%)
C_AP-L_	3.10	90.15	1.11	6.89
C_AP-H_	6.42	86.31	1.55	5.72
C_SBA-15_	0.48	82.32	2.36	14.84
C_CH-L_	0.30	69.46	1.41	28.83
C_CH-H_	0.26	69.18	1.54	29.02

**Table 2:** Textural parameters of C_AP-L_, C_AP-H_, C_SBA-15_, C_CH-L_, and C_CH-H_.

Material	Specific Surface Area (m^2^ g^−1^)	Total Pore Volume (cm^3^ g^−1^)	Average Pore Diameter (nm)
C_AP-L_	107	0.25	9.22
C_AP-H_	101	0.04	1.67
C_SBA-15_	1180	1.17	4.23
C_CH-L_	547	0.47	3.42
C_CH-H_	483	0.42	3.29

**Table 3:** The parameters of Langmuir and Freundlich isotherm models fitted to equilibrium data of losartan adsorption on the surface of functionalized mesoporous carbon materials.

Material	Langmuir			Freundlich		
	q_m_ (mg/g)	K_L_ (L/mg)	R^2^	K_F_ (mg/g (L/mg)^1/n^)	1/n	R^2^
C_AP-L_	98	1.268	0.999	74	0.070	0.814
C_AP-H_	60	0.722	0.999	37	0.121	0.826
C_SBA-15_	162	2.948	0.999	135	0.046	0.933
C_CH-L_	152	1.331	0.999	109	0.086	0.855
C_CH-H_	112	1.450	0.999	82	0.081	0.820

**Table 4:** Parameters of kinetic release models suggesting the mechanism of diclofenac liberation from carbon vehicles.

Material	Zero-Order		First-Order		Higuchi		Hixson–Crowell		Korsmeyer–Peppas		
	k_0_ [h^−1^]	R^2^	k_1_ [h^−1^]	R^2^	k_H_ [h^−1/2^]	R^2^	k_HC_ [h^−1/3^]	R^2^	k_KP_ [h^−n^]	n	R^2^
C_AP-L_	1.797	0.762	0.036	**0.932**	12.455	0.901	0.038	0.871	7.123	0.683	0.758
C_AP-H_	0.824	0.824	0.024	**0.974**	10.316	0.882	0.015	0.696	2.335	0.895	0.899
C_SBA-15_	0.047	0.903	0.001	0.916	0.821	**0.988**	0.001	0.912	1.901	0.369	0.969
C_CH-L_	0.133	**0.971**	0.001	0.891	2.092	0.963	0.002	0.970	0.935	0.112	0.761
C_CH-H_	0.073	0.912	0.001	0.920	1.446	**0.963**	0.001	0.907	0.063	1.060	0.940

## Data Availability

Data available in a publicly accessible repository.
